# Competition Experiments for Legume Infection Identify *Burkholderia phymatum* as a Highly Competitive β-Rhizobium

**DOI:** 10.3389/fmicb.2017.01527

**Published:** 2017-08-15

**Authors:** Martina Lardi, Samanta Bolzan de Campos, Gabriela Purtschert, Leo Eberl, Gabriella Pessi

**Affiliations:** Department of Plant and Microbial Biology, University of Zurich Zurich, Switzerland

**Keywords:** Rhizobium, *Burkholderia*, legume, symbiosis, nodulation, competitiveness

## Abstract

Members of the genus *Burkholderia* (β-proteobacteria) have only recently been shown to be able to establish a nitrogen-fixing symbiosis with several legumes, which is why they are also referred to as β-rhizobia. Therefore, very little is known about the competitiveness of these species to nodulate different legume host plants. In this study, we tested the competitiveness of several *Burkholderia* type strains (*B. diazotrophica*, *B. mimosarum*, *B. phymatum*, *B. sabiae*, *B. symbiotica* and *B. tuberum*) to nodulate four legumes (*Phaseolus vulgaris*, *Macroptilium atropurpureum*, *Vigna unguiculata* and *Mimosa pudica*) under our closely defined growth conditions. The assessment of nodule occupancy of these species on different legume host plants revealed that *B. phymatum* was the most competitive strain in the three papilionoid legumes (bean, cowpea and siratro), while *B. mimosarum* outcompeted the other strains in mimosa. The analysis of phenotypes known to play a role in nodulation competitiveness (motility, exopolysaccharide production) and additional *in vitro* competition assays among β-rhizobial strains suggested that *B. phymatum* has the potential to be a very competitive legume symbiont.

## Introduction

The rhizobia-legume symbiosis is estimated to contribute nearly half of all current biological nitrogen fixation and is an essential element of agricultural sustainability (Vitousek et al., 2002). This symbiosis is manifested by the formation of root nodules on legume plants, in which rhizobia fix atmospheric nitrogen to the benefit of the plant and in exchange for nutrient supply and protection against environmental stresses (Downie, 2010; Oldroyd et al., 2011). Rhizobia are taxonomically diverse and polyphyletic (Masson-Boivin et al., 2009; Sprent et al., 2017). Until recently, all known examples of legume-rhizobial symbiosis were confined to the α-proteobacteria group (Young and Haukka, 1996). This changed with the discovery of nodulating *Burkholderia* and *Cupriavidus* strains, both of which belong to the β-proteobacteria (Chen et al., 2001; Moulin et al., 2001; Bontemps et al., 2010). The number of legume nodulating strains belonging to the environmental clade of the genus *Burkholderia*, for which the new genus *Paraburkholderia* has been proposed recently (Sawana et al., 2014; Beukes et al., 2017), has increased rapidly over the last years (Gyaneshwar et al., 2011; Howieson et al., 2013; Lemaire et al., 2015). These strains of the *Paraburkholderia* clade were shown to nodulate a wide range of legumes, among them several *Mimosa* species (sub-family Mimosoideae) and Papilionoid legumes from different tribes and from several regions of the world (Chen et al., 2003; Chen et al., 2005a; Elliott et al., 2007b; dos Reis et al., 2010; Liu et al., 2012; Mishra et al., 2012; Bournaud et al., 2013). All nodulating and diazotrophic *Burkholderia* species described so far, with the exception of the South African strains *B. tuberum* (Elliott et al., 2007a) and *B. dipogonis* (Liu et al., 2014), were either isolated from or are able to nodulate Mimosa plants (Bournaud et al., 2013). The diversity of mimosoid-nodulating *Burkholderia* is high and includes species such as *B. phymatum*, which is able to nodulate several important legumes including the common bean (*Phaseolus vulgaris*) (Talbi et al., 2010; Moulin et al., 2014). Previous studies have shown that an individual legume plant can be infected by more than one rhizobial strain (Barrett and Parker, 2006; Klonowska et al., 2012; Mishra et al., 2012; Melkonian et al., 2014). For example, *Mimosa* spp. was found to host *Rhizobium* spp., *Burkholderia* and *Cupriavidus* strains (Barrett and Parker, 2006; dos Reis et al., 2010; Gyaneshwar et al., 2011; Bontemps et al., 2016). Two previous studies showed that β-rhizobia are very dominant in the genus *Mimosa* (Elliott et al., 2009; Melkonian et al., 2014). In particular, Elliott et al. (2009) showed that *B. mimosarum* was more competitive than *Cupriavidus taiwanensis* and other tested α-rhizobia on several *Mimosa* species, while Melkonian et al. (2014) reported that *B. phymatum* and *B. tuberum* were more competitive than α-rhizobia on *M. pudica*. In that same study, they also tested the effects of environmental conditions on the competitiveness of *Burkholderia* against other rhizobial species (i.e., *Cupriavidus*) and showed that nitrogen-limited conditions favor the dominance of *Burkholderia* species (Elliott et al., 2009). Other studies showed that β-rhizobial competitiveness was greatly affected by soil pH (Garau et al., 2009; Angus et al., 2013). Beside environmental conditions, competition has been shown to depend on the geographical position and on symbiont and host plant genetic diversity (Dowling and Broughton, 1986; Mishra et al., 2012; Melkonian et al., 2014; Bontemps et al., 2016). For the well-studied rhizobia from the α-subclass of proteobacteria, several phenotypic traits were shown to be important for their nodulation competitiveness (Triplett and Sadowsky, 1992). These included motility (Mellor et al., 1987; Liu et al., 1989), exopolysaccharide (EPS) and lipopolysaccharide (LPS) production (Bhagwat et al., 1991; Zdor and Pueppke, 1991; Geddes et al., 2014), antibiotic production (Schwinghamer and Belkengren, 1968; Robleto et al., 1998) and the catabolism of certain compounds such as proline, *myo*-inositol, and glycerol (Jiménez-Zurdo et al., 1995; Fry et al., 2001; Kohler et al., 2010; Ding et al., 2012).

In this work, we aimed to study the competition among β-rhizobial strains of the genus *Burkholderia* using – in addition to the well-studied β-rhizobial plant host *Mimosa pudica* – three papilionoid legumes of major economic importance in agriculture: *P. vulgaris* (bean), *Macroptilium atropurpureum* (siratro) and *Vigna unguiculata* (cowpea). We used sterile vermiculite and constant growth conditions to investigate the competitiveness of several different *Burkholderia* type strains (*B. diazotrophica* LMG26031, *B. mimosarum* LMG23256, *B. phymatum* LMG21445, *B. sabiae* LMG24235, *B. symbiotica* LMG26032, *B. tuberum* LMG21444) to nodulate a single legume. Seeds from four different host plants (*M. pudica*, bean, cowpea and siratro) were inoculated with a bacterial mixture and after 3 or 4 weeks, the nodule occupancy rates of each species on different legumes were assessed by employing specific PCRs and *recA* sequencing. *B. phymatum* revealed to be the most competitive strain in nodulating the roots of three of the four tested legumes under the closely defined growth conditions that we used. *B. mimosarum* is shown to outcompete the other strains in *M. pudica*. The assessment of traits important for competitiveness provided further support that *B. phymatum* is very competitive, as the strain (i) was among the most motile strains, (ii) produced more EPSs compared to the other strains, and (iii) outcompeted the other strains *in vitro*.

## Materials and Methods

### Bacterial Strains, Plasmids and Growth Conditions

The bacterial strains, plasmids and primers employed in this work are listed in Supplementary Table S1. *Burkholderia* strains were cultivated under aerobic conditions at 30°C in LB medium without salt (10 g of tryptone and 5 g yeast extract per liter). Bacterial cultures for competition plant tests were washed in defined buffered AB-minimal medium (Clark and Maaloe, 1967) without nitrogen and with 10 mM sodium citrate as the carbon source, while for phenotypical tests they were washed in LB medium without salt. Growth of the *Burkholderia* strains on plates was assessed in AB-minimal medium (Clark and Maaloe, 1967) containing 10 mM glucose, 15 mM succinic acid or 10 mM sodium citrate as carbon source; PIA (*Pseudomonas* Isolation Agar, Difco) and in LB medium without salt. To assess liquid growth in LB without salt, cultures were grown at 30°C with agitation (220 rpm) in 250 ml Erlenmeyer flasks containing 100 ml of medium. Independent duplicates for all the bacterial strains were tested.

### Plant Growth Conditions

Common bean seedlings (*Phaseolus vulgaris*, cv. *Negro jamapa*; kindly provided by Professor Eulogio Bedmar, Granada, Spain) were surface sterilized as previously described (Talbi et al., 2010). *Macroptilium atropurpureum* (siratro), *Vigna unguiculata* (cowpea) and *Mimosa pudica* (mimosa) were surface sterilized as described previously (Klonowska et al., 2012). Seeds were subsequently deposited on 0.8% agar plates and incubated in the dark at 30°C. After one and a half days, germinated seedlings were planted into 250 ml autoclaved yogurt-jars containing vermiculite (VTT-Group, Muttenz, Switzerland) and 170 ml diluted Jensen medium (Hahn and Hennecke, 1984). The pH in the pots was 6.5. Direct seed inoculation with the individual bacterial strains or with the specific bacterial mixtures was performed. The plants were grown with the following parameters: temperature, 22°C at night and 25°C during the day; light, approximately 16 h (200 μMol intensity); humidity 60%. The plants were harvested 21 days post infection (dpi) for bean and cowpea or 28 dpi for siratro and mimosa.

### Determination of Symbiotic Effectiveness

Nodule number and nodule dry weight were determined as described previously (Göttfert et al., 1990). For the determination of the shoot dry weight and N content, six plants for each of the bacteria–plant combinations tested were dried at 60°C for 72 h. Shoots were first weighed, crushed in a mortar and placed in one or more 2 ml Eppendorf. Each sample was homogenized using a Tissuelyser (Qiagen, Hilden, Germany) and a glass bead of 7 mm diameter (Assistent, Rhön, Germany) for 2 min at 30 Hertz. After homogenization, 1.5–2 mg of the samples were taken for the determination of the total N content using LECO TruSpec CHM micro instrument (LECO Corporation, St. Joseph, MI, United States), which is based on the Dumas or combustion method.

### Competition Assays *In Planta*

The strains were grown in 4 ml LB medium without salt until an optical density (OD_600_) of 2. At this optical density, all strains were in the same growth phase (end of exponential phase). After washing the cultures twice in minimal medium without nitrogen, the OD_600_ of each culture was adjusted to 0.01 and subsequently diluted 1000-fold. This dilution was plated to ensure that the same amount of cells was used for each strain in the mix. The bacterial mixture containing around 10^2^ colony forming units (CFU) each of *B. diazotrophica*, *B. mimosarum*, *B. phymatum*, *B. sabiae* and *B. tuberum* was immediately applied on bean and siratro germinated seeds (Mix 1). The same mix excluding *B. tuberum* was used for cowpea (Mix 2). Mimosa seeds were inoculated with 10^2^ CFU each of *B. diazotrophica*, *B. mimosarum*, *B. phymatum* and *B. symbiotica* (Mix 3). The bacterial mixtures were prepared in equal volumes of cell suspension (1 ml/seedling). The plants in sterilized vermiculite were grown as described above and regularly watered with deionized water. Two independent experiments were performed and per experiment 4–6 plants for bean, cowpea, mimosa and siratro were analyzed. After 21 dpi (for bean and cowpea) or 28 dpi (for siratro and mimosa), roots were surface sterilized by soaking for 10 s in 100% ethanol, followed by 3 min in 2.5% sodium hypochlorite and then washed 5 times with sterile deionized water (Vincent, 1970). All the nodules present on the root of each plant were individually collected in an Eppendorf containing 100 μl LB without salt and 100 μl 50% glycerol. Afterward, each nodule of one plant was crushed and the extract was plated onto solid 0.06% YEM medium. Single colonies and 2 μl from the nodule extract were tested by PCR using specific primers (Supplementary Table S1): for *B. phymatum* (Bphy_comp_F, TGCGCTGCTTTCCATTTCAC and Bphy_comp_R, AGTAGTCGCTGCTATCGTGC), for *B. mimosarum* (Bmim_comp_F, GCACTTTACGTCCAGACACG and Bmim_comp_R, CGTCGTGAGTCAGGTAACCA) and for *B. tuberum* (Btub_comp_F, GCCGAACTAGGATTGTACGC and Btub_comp_R, CGCGAACTCCAGACACAATA). If no product was obtained, PCR were performed with *Burkholderia* degenerate *recA* primers (recABurk1_F, GATCGARAAGCAGTTCGGCAA and recABurk1_R, TTGTCCTTGCCCTGRCCGAT), which amplified a product with all the six *Burkholderia* strains used (Mishra et al., 2012). To identify the strains specifically, the obtained PCR product was purified and sequenced (at Microsynth AG, Switzerland). Further proof for all the nodules infected by *B. tuberum* was obtained by plating the nodule extract on PIA plates, a medium that allows selective growth of *B. tuberum*. Nodules obtained from the second plant competition test were directly tested by PCR from the crushed material. The nodule occupancy rate for each legume was calculated by dividing the number of nodules occupied by one strain by the total number of nodules analyzed and present on the roots.

### Phenotypical Analysis Including Competition Assays *In Vitro*

Exopolysaccharide production was visualized on modified YEM medium plates (1% mannitol, 0.06% yeast extract) (Zlosnik et al., 2008). Plates were incubated for 4 days at 30°C. Swimming motility was tested by inoculating cells onto plates containing LB without salt supplemented with 0.1% casamino acids that were solidified with 0.2% agar (Eberl et al., 1996; Lardi et al., 2015). Plates were incubated for 30 h at 30°C. Competition experiments on plate (*in vitro*) were performed in 50:50 ratio for *B. phymatum* against *B. tuberum*, *B. mimosarum* against *B. tuberum* and *B. mimosarum* against *B. phymatum* GFP-tagged (Elliott et al., 2007b). Cultures were grown o/n in LB medium without salt until an OD_600_ of 3 and washed once with LB without salt. The inoculum was normalized at OD_600_ = 0.1 (corresponding to a CFU of 10^8^). The bacteria of interest were equally mixed in a 50:50 volume and 5 μl were spotted onto an LB plate without salt and incubated for 24 h at 30°C. The cells were recollected in 1 ml of LB without salt and CFU were counted on LB without salt plates and onto selective plates, i.e., PIA for *B. tuberum* and LB medium without salt containing tetracycline for *B. phymatum* GFP.

### Statistical Analyses

Swimming motility was statistically analyzed using unpaired *t*-test (^∗∗∗∗^*p* < 0.0001, ^∗∗∗^*p* < 0.001, ^∗∗^*p* < 0.01 and ^∗^*p* < 0.05). The analyses were performed using GraphPad Prism software version 7.0c. For the competition assay *in vitro* standard deviation (SD) was calculated and inserted into the histograms. For determination of dry weight, N content and nodule dry weight ANOVA and Tukey’s test were performed. The correlation between N content, nodule dry weight and nodule number was analyzed with GraphPad Prism software version 7.0c.

## Results

### Growth and Nodulation Behavior of the *Burkholderia* Strains Used in Competition

Before setting up the competition experiments, the growth behavior of the different *Burkholderia* strains, i.e., *B. diazotrophica* [originally isolated from *Mimosa candollei*; (Sheu et al., 2013)], *B. mimosarum* [originally isolated from *Mimosa pigra*, (Chen et al., 2006)], *B. phymatum* [isolated from *Mimosa* species (Elliott et al., 2007b)], *B. sabiae* [originally isolated from *Mimosa caesalpiniifolia*, (Chen et al., 2008)], *B. symbiotica* [originally isolated from *Mimosa cordistipula*, (Sheu et al., 2012)] and *B. tuberum* [originally isolated from *Aspalathus carnosa*, (Moulin et al., 2001; Vandamme et al., 2002)], was tested in five different media (**Table 1**). All six strains were able to grow within 48 h in LB without salt and in AB minimal medium with succinic acid as carbon source, with the exception of *B. symbiotica* that needed 6 days to grow on AB minimal medium with succinic acid. When AB minimal medium was supplemented with sodium citrate as carbon source, *B. diazotrophica* and *B. symbiotica* were not able to grow. Additionally, *B. symbiotica* could not use glucose as carbon source. *B. tuberum* was the only strain able to grow on PIA, which contains a broad-spectrum antimicrobial (Irgasan) that is commonly used to select for *Burkholderia* strains (Kost et al., 2014).

**Table 1:** Growth of the six *Burkholderia* type strains in different complex and minimal media.

	Bacterial strains					
Growth media^a^	*B. diazotrophica* LMG26031	*B. mimosarum* LMG23256	*B. phymatum* LMG21445	*B. sabiae* LMG24235	*B. symbiotica* LMG26032	*B. tuberum* LMG21444
LB without salt	+++	+++	+++	+++	+++	+++
AB – glucose	++	+++	+++	+++	-	+++
AB – sodium citrate	-	+++	++	++	-	+
AB – succinic acid	+++	+++	+++	+++	+	+++
PIA (*Pseudomonas* Isolation Agar)	-	-	-	-	-	+++

*^a^Growth was assessed by visual inspection after incubation at 30°C; 3 independent replicates were performed. “+++”: single colonies after 48 h; “++”: single colonies after 4 days; “+”: single colonies after 6 days; “-”: no growth.*

A growth kinetic analysis in LB liquid medium without salt over 25 h showed that all strains displayed similar growth rates and reached the stationary phase at an OD_600_ of approximately 3 (Supplementary Figure S1).

Next, we tested nodulation for each of the overall 24 different *Burkholderia*-legume combinations (**Table 2**). *B. diazotrophica*, *B. mimosarum* and *B. phymatum* were able to nodulate all the four tested legumes, i.e., *M. pudica* (tribe Mimoseae), *P. vulgaris* (bean; tribe Phaseoleae), *M. atropurpureum* (siratro; tribe Phaseoleae) and *V. unguiculata* (cowpea; tribe Phaseoleae). *B. sabiae* formed nodules on all legumes except on mimosa. While *B. tuberum* induced nodules in bean and siratro, *B. symbiotica* was the most host-specific strain tested and only able to nodulate mimosa.

**Table 2:** Tested legumes and bacterial mixes employed.

	Bacterial strains					
Legumes	*B. diazotrophica* LMG26031	*B. mimosarum* LMG23256	*B. phymatum* LMG21445	*B. sabiae* LMG24235	*B. symbiotica* LMG26032	*B. tuberum* LMG21444
**Bean** *Phaseolus vulgaris* *Negro jamapa*^ab^	+	+	+	+	-	+
**Cowpea** *Vigna unguiculata* (L.) Walp cv. Red Caloona^c^	+	+	+	+	-	-
**Mimosa** *Mimosa pudica* Samen Mauser AG^d^	+	+	+	-	+	-
**Siratro** *Macroptilium atropurpureum***** Sunshine seeds^d^	+	+	+	+	-	+

*^a^Biovar of the seeds. ^b^Kindly provided by Professor Eulogio Bedmar (Granada, Spain) and subsequently produced in our lab. ^c^Kindly provided by Hans-Martin Fischer lab from ETH (Zurich, Switzerland) and subsequently produced in our lab. ^d^Producer of the commercial seeds.*

### Capacity of Different *Burkholderia* Strains to Compete for Nodulation of Different Legumes

**FIGURE 1:**
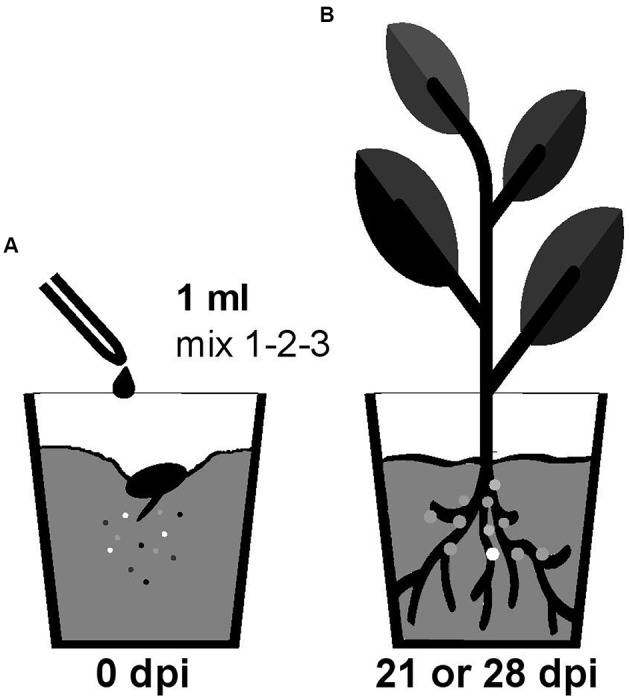
Workflow of the competition experiment *in planta*. Germinated seedlings were inoculated with 1 ml of bacterial mix (mix 1 for siratro and bean; mix 2 for cowpea and mix 3 for mimosa) **(A)**. After 21 (bean and cowpea) or 28 (mimosa and siratro) days all the bacteria present within nodules of the root apparatus were identified by PCR with degenerate primers and sequencing **(B)**. The different colors represent the different *Burkholderia* type strains present in the mix **(A)** and inside the nodules **(B)**.

**FIGURE 2:**
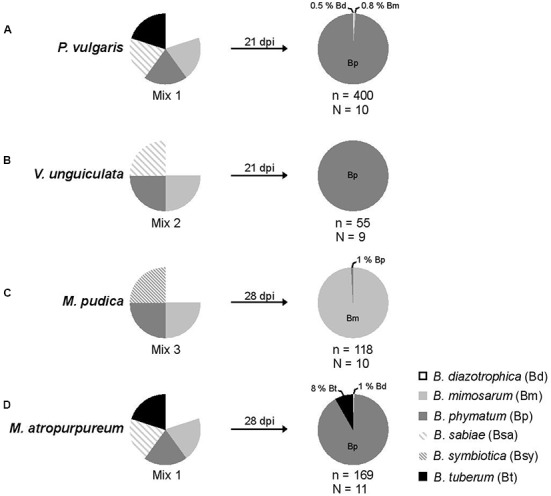
Relative nodule occupancy of several nodulating and nitrogen-fixing *Burkholderia* strains in four legumes. For each tested legume, the ratio of each bacteria in the initial inoculum is shown followed by the ratio obtained 21 (bean and cowpea) or 28 (mimosa and siratro) days post infection (dpi). A mixture of five *Burkholderia* strains – *B. diazotrophica*, *B. mimosarum*, *B. phymatum*, *B. sabiae*, and *B. tuberum* (mix 1) – was inoculated on bean (*P. vulgaris*) **(A)** and siratro (*M. atropurpureum*) **(D)**. The same bacterial mixture excluding *B. tuberum* (mix 2) was applied onto cowpea (*V. unguiculata*) **(B)**. For mimosa (*M. pudica*) **(C)** four *Burkholderia* strains were inoculated: *B. diazotrophica*, *B. mimosarum*, *B. phymatum* and *B. symbiotica* (mix 3). “N” indicates the number of analyzed plants and “n” the number of analyzed nodules.

Nodulation competitiveness of *B. diazotrophica*, *B. mimosarum*, *B. phymatum*, *B. sabiae*, *B. symbiotica* and *B. tuberum* was determined by competition experiments using different host plants. Importantly, we performed all experiments by growing the plants in sterile vermiculite and under our own closely defined growth conditions (see Materials and Methods and **Figure 1**). Commercial seeds from the mimosoid legume *M. pudica* were inoculated with a 1 ml mixture containing around 100 cells (Supplementary Figure S2) of the following strains, which we had shown before (s. above) to be able to nodulate *M. pudica*: *B. diazotrophica*, *B. mimosarum*, *B. phymatum* and *B. symbiotica* (mix 3) (**Figure 2** and **Table 2**). *V. unguiculata* seeds were infected with a mixture containing *B. diazotrophica*, *B. mimosarum*, *B. phymatum* and *B. sabiae* (mix 2) (**Figure 2** and **Table 2**). The same mix with *B. diazotrophica*, *B. mimosarum*, *B. phymatum*, *B. sabiae* and in addition *B. tuberum* (mix 1) was used to inoculate the commercial seeds of *M. atropurpureum* and the seeds of *P. vulgaris* (**Figure 2** and **Table 2**).

In order to provide the best possible analysis of the nodule occupancy, we decided to harvest and individually assess each and every single root nodule (typically ranging from a minimum of 2 in *V. unguiculata* to a maximum of 47 in *P. vulgaris*) per plant. After 3 or 4 weeks all nodules from one plant were collected and subsequently analyzed for occupancy using strain-specific PCRs (see Materials and Methods section for details). The identity of the strains was verified by amplifying and sequencing the *recA* gene (Mishra et al., 2012). The results showed that bean was preferentially nodulated by *B. phymatum* (99% of the nodules), and only 2 and 3 nodules out of 400 analyzed were occupied by *B. diazotrophica* and *B. mimosarum*, respectively (**Figure 2A**). While *B. phymatum* was the most competitive strain on siratro (91% nodule occupancy), 8% of the siratro nodules (14) contained *B. tuberum*, and one nodule was occupied by *B. diazotrophica* (**Figure 2D**). The nodule occupancy of *B. phymatum* in cowpea was 100% in all the 9 analyzed plants (55 nodules overall) (**Figure 2B**). In mimosa *B. mimosarum* occupied 99% of the nodules. Only one nodule out of 118 analyzed nodules was occupied by *B. phymatum* (**Figure 2C**). Using this strategy, we did not identify any nodules colonized by two species (mixed nodules).

### Competitiveness for Nodulation versus Symbiotic Performance

The performance of a single strain inoculated in a single legume was assessed by recording shoot dry mass, shoot N content, nodule number per plant and dry weight per nodule (**Table 3**). We tried all legumes in combinations with the four most competitive strains – *B. diazotrophica*, *B. mimosarum*, *B. phymatum* and *B. tuberum* – (**Table 3**) by using the same plant growth conditions we applied for competition experiments (see Materials and Methods). Competition experiments on bean revealed that *B. phymatum* is the most competitive strain and only few nodules were colonized by *B. mimosarum* and *B. diazotrophica* (s. above). The symbiotic efficiency on bean differed depending on the inoculated strain. Plants inoculated with *B. mimosarum* showed a higher shoot N content when compared with the other three strains tested (**Table 3**). Bean plants infected with *B. tuberum* also displayed a higher shoot dry weight and N content compared to the most competitive strain *B. phymatum*. Inoculation of single strains on cowpea revealed that the otherwise best competitor *B. phymatum* was not performing well in this host plant. In fact, the shoot dry weight and the N content of plants inoculated with *B. phymatum* was similar to that measured in uninoculated control plants (**Table 3**). In *M. pudica*, *B. mimosarum* was not only outcompeting the other strains but also performed best in terms of shoot dry weight and provided N content (**Table 3**). Based on the determination of N content, the most efficient strains in providing nitrogen to siratro plants were *B. diazotrophica* and *B. tuberum* (**Table 3**). Although to a lesser extent than *B. phymatum*, *B. tuberum* was able to colonize few nodules in competition with the other bacterial strains (**Figure 2**). In contrast, the best competitor in siratro, *B. phymatum*, was less efficient in providing nitrogen to host plants compared to *B. diazotrophica* and *B. tuberum* when inoculated alone.

**Table 3:** Symbiotic properties of the four most competitive *Burkholderia* type strains.

	Bacterial strains				
Parameter	Uninoculated control	*B. diazotrophica* LMG26031	*B. mimosarum* LMG23256	*B. phymatum* LMG21445	*B. tuberum* LMG21444
**Total shoot N content [mg N/g shoot dry weight]**					
Bean	11.09a	14.97ac	**22.59**bc	15.33ac	16.32ac
Cowpea	11.32a	11.82a	**13.60**ab	9.67ac	ND
Mimosa	11.20a	19.32b	**29.59**c	20.20b	ND
Siratro	10.60a	**20.39**b	8.35a	12.27ac	19.05bc
**Dry weight shoot [mg shoot]**					
Bean	369.05a	404.40a	383.47a	374.83a	**407.75**a
Cowpea	83.45a	89.78a	**99.37**a	81.28a	ND
Mimosa	13.80a	21.32a	**41.35**a	27.22a	ND
Siratro	29.20a	31.48a	32.03a	**35.50**a	31.12a
**Dry weight nodules [mg]**					
Bean	0	0.81a	**1.10**b	0.42c	0.56ac
Cowpea	0	0.12a	**0.20**a	0.15a	ND
Mimosa	0	0.13a	**0.14**a	0.11a	ND
Siratro	0	**0.41**a	0.29a	0.31a	0.35a
**Nodule number**					
Bean	0	58.60a	**97.00**b	82.50b	49.17a
Cowpea	0	9.67a	4.50ab	**12.50**ac	ND
Mimosa	0	**12.56**a	9.50a	7.82a	ND
Siratro	0	5.33a	7.67ab	13.00b	**14.00**b

*For each row, values with the same letter are not significantly different (ANOVA, Tukey’s test with p-value ≤ 0.05). ND, not determined since this bacterial strain is not nodulating this host plant. In bold the best strain for each row is indicated*.

For some *Burkholderia* – legume combinations we observed a positive correlation between shoot N contents and nodule dry weight and a negative correlation between shoot N contents and number of nodules. For instance, in siratro *B. diazotrophica* produced the highest shoot N content and nodule dry weight, but showed the lowest number of nodules. In cowpea*, B. mimosarum* had the same effect. Indeed, the legume usually regulates the number of nodules according to their activity, which is proportional to their size (Ferguson et al., 2010). In the case of common bean, that was shown to have a relaxed autoregulation of nodulation (AON) (George and Robert, 1991) no negative correlation between N content and nodule number was observed.

### Phenotypic Analysis of the *Burkholderia* Strains Used in Competition

**FIGURE 3:**
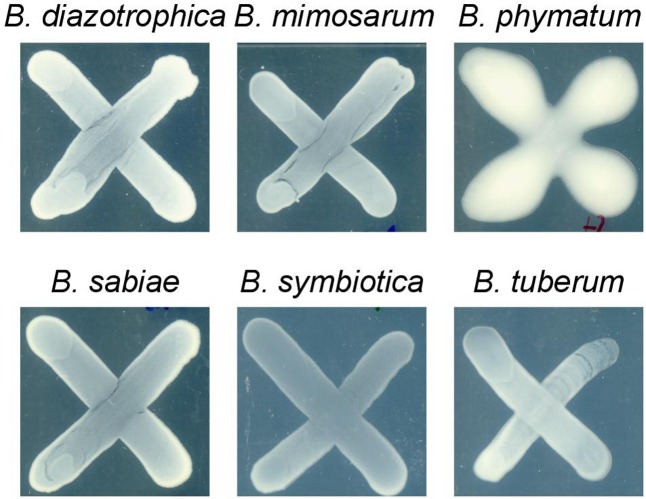
Exopolysaccharides (EPS) production in six tested *Burkholderia* strains. The plates were supplemented with 0.06% of yeast extract and incubated for 4 days. At least 3 independent replicates were performed per each strain.

**FIGURE 4:**
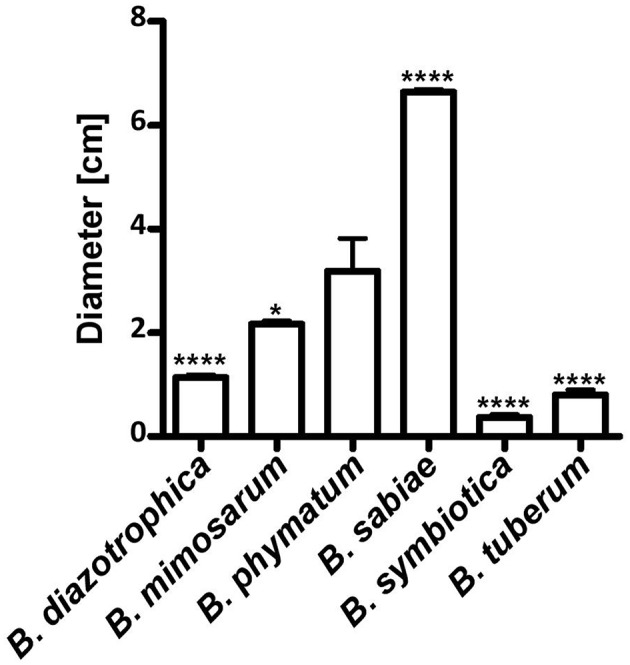
Motility of six nodulating and nitrogen-fixing *Burkholderia* type strains. Swimming motility on LB without salt plates was measured after 30 h incubation. Error bars indicate SD. At least 3 independent replicates were performed. Asterisks indicate a significant difference between *B. phymatum* and the indicated strain (^∗∗∗∗^*p*-value < 0.0001; ^∗^*p*-value < 0.05; unpaired two-tailed *t*-test).

Two phenotypes known to be relevant for nodulation competitiveness in α-Rhizobia were assessed (Triplett and Sadowsky, 1992): EPS production and motility. **Figure 3** shows that on an agar plate containing mannitol the most competitive strain on bean, cowpea and siratro, *B. phymatum*, was more mucoid compared to the other five strains, suggesting an increased EPS production. This difference among the tested strains is visible even after a longer incubation of the plates (6 days). In addition, *B. phymatum* was also able to swim; in fact, it was the second-best swimmer after *B. sabiae* (**Figure 4**). While *B. mimosarum*’s swimming ability was about one third of that of *B. sabiae*, the other three strains – *B. diazotrophica*, *B. symbiotica* and *B. tuberum* – were drastically impaired in their swimming capabilities compared to *B. sabiae*.

**FIGURE 5:**
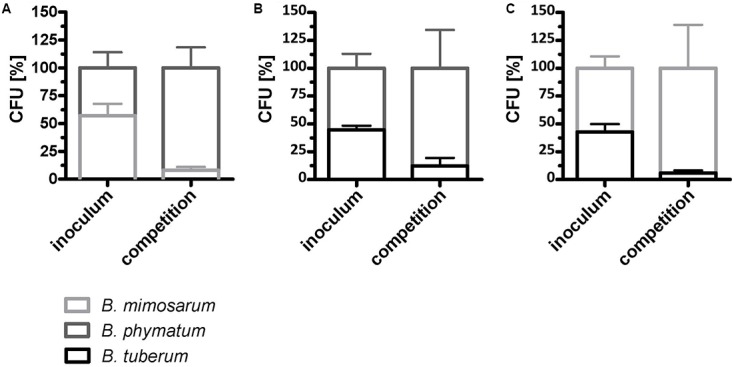
Rhizobial competition on plate. A mixture of *B. phymatum* GFP-tagged against *B. mimosarum* **(A)**, *B. phymatum* against *B. tuberum* **(B)** and of *B. mimosarum* against *B. tuberum* **(C)** was tested for competition for 24 h on plate. The percentage of colony forming units (CFU) of the two competing strains in the inoculum (inoculum) and recovered after 24 h of contact (competition) is shown. Error bars indicate standard deviation (SD). At least 3 independent replicates were analyzed.

We next evaluated if the competition occurs already under free-living conditions. For this we mixed two strains in equal amount and incubated them on an agar plate for 24 h. As a control, each strain was spotted alone on the plate and was incubated for the same time. The following combinations were tested: *B. phymatum* – *B. mimosarum*, *B. phymatum* – *B. tuberum* and *B. mimosarum – B. tuberum* (**Figure 5**). In the first combination, *B. phymatum* was outcompeting *B. mimosarum* and after 24 h only 8% of the cells were *B. mimosarum* (**Figure 5A** and Supplementary Figure S3A). When competing *B. phymatum* against *B. tuberum*, we recovered about 88% *B. phymatum* and 12% *B. tuberum* (**Figure 5B** and Supplementary Figure S3B). The last combination tested revealed that *B. mimosarum* outcompeted *B. tuberum* (94% against 6% of recovered cells) (**Figure 5C** and Supplementary Figure S3C). Interestingly, while the amount of *B. phymatum* cells growing alone or as a mixture remained constant after 24 h, the number of cells of the outcompeted strain (*B. mimosarum* or *B. tuberum*) was always around 10-fold lower after competition (Supplementary Figure S3).

## Discussion

Several *Burkholderia* strains (β-rhizobia) have recently been isolated from root nodules and were found to efficiently nodulate a range of legumes belonging to the Mimosoideae [*Mimosa* spp., (Elliott et al., 2007b)] and to the Papilionoideae (Elliott et al., 2007a) subfamilies. *B. phymatum* has been shown to be highly promiscuous with many mimosoid legumes (Moulin et al., 2014) and in fynbos species (Lemaire et al., 2016). Several legumes have been shown to associate with both α- and β-rhizobia (Barrett and Parker, 2006; Elliott et al., 2007a; Gyaneshwar et al., 2011; Klonowska et al., 2012) and often display a higher affinity for α- or β-rhizobia depending on their geographical origin (Melkonian et al., 2014; Bontemps et al., 2016). Trapping experiments with different *Mimosa* species recovered mainly strains belonging to the *Burkholderia* genus (Barrett and Parker, 2005; Chen et al., 2005a,b; Parker et al., 2007; Bontemps et al., 2010; dos Reis et al., 2010; Liu et al., 2012; Mishra et al., 2012) and competition experiments showed that *Burkholderia* strains outcompete *C. taiwanensis* and α-rhizobia on the three major invasive *Mimosa* species *M. diplotricha*, *M. pigra* and *M. pudica* (Elliott et al., 2009). Among the *Burkholderia* strains, *B. phymatum* was shown to be very competitive on *Mimosa* independently of the geographical origin of the seeds (Melkonian et al., 2014). Nodulation competitiveness in the Rhizobium-legume symbiosis is a complex process. In the field, it depends at least on three factors, namely the rhizobial genome, the legume genome and the environment. To investigate the role of genomic factors of both the host plant and its rhizobial partner in the symbiosis, we have established and used a particular and defined environment throughout all our experiments (inoculation of a germ-free seed with a defined mixture of β-rhizobial strains, plant grown in sterile vermiculite under constant growth conditions). We competed several nodulating and nitrogen fixing *Burkholderia* type strains using four different legumes belonging to two sub-families: *M. pudica* from the Mimosoideae and *P. vulgaris* (bean), *M. atropurpureum* (siratro) and *V. unguiculata* (cowpea) from the Papilionoideae. *P. vulgaris* and *V. unguiculata* are major grain legumes relevant for agricultural sustainability worldwide. *V. unguiculata* can also be used as forage plant and *M. atropurpureum* is a perennial tropical forage legume^1^. We show here for the first time that *B. phymatum* was dominating over the other five strains in the nodules of the three papilionoid legumes bean, cowpea and siratro. *B. mimosarum* revealed to be the most competitive strain on *M. pudica*. The dominance of *B. mimosarum* over *B. phymatum* on *M. pudica* confirms the data reported by Liu et al. (2012), which were sampling *M. pudica* nodules from various locations in China. However, these *B. mimosarum* strains were shown to harbor *nodA* from *B. phymatum*. In contrast, two other studies performed on *M. pudica* grown in sterile conditions (Elliott et al., 2009; Melkonian et al., 2014) showed that *B. phymatum* was more competitive than *B. mimosarum*. The presence of more strains in our rhizobial mixture used to inoculate the plants, the origin of the seeds and the different growth conditions could possibly explain the different outcomes of the experiments.

While *B. phymatum* has so far only been isolated from one location in its native South America (Mishra et al., 2012), it is quite widespread in association with invasive *Mimosa* species in Asia Elliott et al., 2007b; Liu et al., 2011; Gehlot et al., 2013) and is also found in common beans cultivated in Morocco (Talbi et al., 2010). *B. mimosarum* on the other hand is very widespread in South America (Chen et al., 2005a; Bontemps et al., 2010; Mishra et al., 2012) and Asia (Chen et al., 2005b; Elliott et al., 2009; Liu et al., 2012; Gehlot et al., 2013).

Our results suggested that the competitiveness of *B. phymatum* is not always proportional to its symbiotic performance. In fact, while *B. phymatum* was the most competitive strain on the three papilionoid legumes, *B. mimosarum* was the most efficient strain on bean and cowpea in terms of N content and nodule dry weight. Siratro plants were performing better when inoculated with *B. diazotrophica* and *B. tuberum*. This suggests that all three papilionoid legumes (bean, cowpea and siratro) do not favor those rhizobia that would supply them with a higher amount of nitrogen. This is not the case with *M. pudica*, which seems to promote association with the strain *B. mimosarum*, which is providing the highest shoot N content and nodule dry weight (**Figure 2** and **Table 3**).

The mechanisms that confer a competitive advantage to *Burkholderia* strain for nodulation are not known. From the rhizobial side, it has been shown for α-rhizobia that cell-surface structures such as LPS (Bhagwat et al., 1991; Lagares et al., 1992) and EPS (Zdor and Pueppke, 1991; Geddes et al., 2014) are important for competition. Moreover, in *Bradyrhizobium diazoefficiens* it has been reported that a flagella mutant was less competitive than the wild type for legume nodulation suggesting that motility of the strain is an important phenotype for a successful competition (Liu et al., 1989; Althabegoiti et al., 2011). Interestingly, *B. phymatum* was the strain producing more EPS on mannitol plates (**Figure 4**), suggesting that this phenotype may contribute to the nodulation competitiveness of the strain. Inspection of *B. phymatum* genome revealed the presence of a potential EPS cluster, which shares homologies with the cepacian cluster identified in *B. cenocepacia* strain H111 (Ferreira et al., 2010; Moulin et al., 2014). Preliminary tests suggested that, similar to the situation in *B. cenocepacia* (Lardi et al., 2015), the expression of the *B. phymatum* EPS cluster is induced in nitrogen limiting conditions, which are known to be important to establish a successful infection (Lardi et al., unpublished data). Additionally, the assessment of motility behavior showed that *B. phymatum* was the second-best motile strain (**Figure 5**). As described above, this trait may confer an additional competitive advantage to *B. phymatum*. However, since direct seed inoculation was performed, motility may not be an essential trait in our setup.

Competition test performed *in vitro* suggest that *B. phymatum* is able to inhibit other *Burkholderia* strains such as *B. mimosarum* and *B. tuberum* also on plates. Although a much higher amount of cells as well as a lower volume have been used for this *in vitro* competition assay (performed with two bacterial species) compared to the *in planta* competition test, the results suggest that rhizobia-rhizobia inhibition may also influence the outcome of the competition for nodulation. Several mechanisms are known to underlie growth inhibition including the contact-dependent growth inhibition (CDI) system, which is based on two-partner secretion proteins that suppress the growth of neighboring cells unless the recipient bacterium produces a corresponding immunity protein (Aoki et al., 2010). This system is widely distributed in the genus *Burkholderia* (Nikolakakis et al., 2012; Garcia et al., 2016). Growth inhibition can also be mediated via a type VI secretion system (T6SS), which is similar to a phage tail spike and can penetrate adjacent eukaryotic or prokaryotic target cells and deliver toxic effector proteins that can cause contact-dependent killing. A T6SS was first identified in the α-rhizobial strain *Rhizobium leguminosarum* (Bladergroen et al., 2003) and shown to be important for nodulation of *Pisum sativum* (pea). Many Gram-negative bacteria use this secretion system for killing competitors (Mougous et al., 2006; Pukatzki et al., 2006; Schwarz et al., 2010; Bernal et al., 2017). Both the CDI system and the type VI secretion system are present in *B. phymatum* genome (Moulin et al., 2014). Another possibility to explain this growth inhibition phenotype could be that *B. phymatum* produces a compound, which is inhibitory for the growth of other *Burkholderia* strains present in the mixture. Additional experiments will be required to investigate the possible contribution of these traits to the exceptional competitiveness of *B. phymatum*.

In the future, it would be interesting to change our standard experimental settings and analyze the shift in competitiveness between β-rhizobial symbionts as well as perform competition experiments between selected α- and β-rhizobia to identify a competitive Rhizobium strain, which could be used as inoculant providing these agriculturally important legumes with high levels of nitrogen.

## Author Contributions

Conceived and designed the experiments: ML and GP. Performed the experiments: ML, GPu, and SdC. Analyzed the data: ML, SdC, LE, and GP. Wrote the paper: ML and GP.

## Conflict of Interest Statement

The authors declare that the research was conducted in the absence of any commercial or financial relationships that could be construed as a potential conflict of interest.

## Footnotes

1 http://www.feedipedia.org/
