# Supplementary material for: Competition Experiments for Legume Infection Identify Burkholderia phymatum as a Highly Competitive β-Rhizobium
Source: Front Microbiol. 2017 Aug 15;8:1527. doi: 10.3389/fmicb.2017.01527 (PMC5559654; doi:10.3389/fmicb.2017.01527)
Supplement: Supplementary file 1 [file Table_1.docx]

# **Table S1:** Bacterial strains and oligonucleotides used in this study.

| Strain or Oligonucleotide | Description | Reference |
| --- | --- | --- |
| Strains |  |  |
| *B. diazotrophica* LMG26031 | Wild-type strain isolated from *Mimosa* spp | (Sheu et al., 2013) |
| *B. mimosarum* LMG23256 | Wild-type strain isolated from *Mimosa* spp | (Chen et al., 2006) |
| *B. phymatum* LMG21445 | Wild-type strain isolated from  *Mimosa* spp | (Elliott et al., 2007) |
| *B. sabiae* LMG24235 | Wild-type strain isolated from *Mimosa caesalpiniifolia* | (Chen et al., 2008) |
| *B. symbiotica* LMG26032 | Wild-type strain isolated from *Mimosa* spp | (Sheu et al., 2012) |
| *B. tuberum* LMG21444 | Wild-type strain isolated from *Aspalathus carnosa* | (Vandamme et al., 2002) |
| *B. phymatum* STM815GFP | Wild-type strain containing GFP inserted with Tn5 method;Tc^R^ | (Elliott et al., 2007) |
|  |  |  |
| Oligonucleotides |  |  |
|  |  |  |
| Bphy_comp_F | TGCGCTGCTTTCCATTTCAC | This study |
| Bphy_comp_R | AGTAGTCGCTGCTATCGTGC | This study |
| Bmim_comp_F | GCACTTTACGTCCAGACACG | This study |
| Bmim_comp_R | CGTCGTGAGTCAGGTAACCA | This study |
| Btub_comp_F | GCCGAACTAGGATTGTACGC | This study |
| Btub_comp_R | CGCGAACTCCAGACACAATA | This study |
| Bdia_comp_F | ACGAACTGCCTCGTCATCTT | This study |
| Bdia_comp_R | CGACACCTTGTTCTTGACGA | This study |
| recABurk1_F | GATCGARAAGCAGTTCGGCAA | (Mishra et al., 2012) |
| recABurk1_R | TTGTCCTTGCCCTGRCCGAT | (Mishra et al., 2012) |

**References**

Chen, W. M., de Farja, S. M., Chou, J. H., James, E. K., Elliott, G. N., Sprent, J. I., et al. (2008). *Burkholderia sabiae* sp. nov., isolated from root nodules of *Mimosa caesalpiniifolia*. *Int. J. Syst. Evol. Microbiol.* 58, 2174–2179. doi:10.1099/ijs.0.65816-0.

Chen, W. M., James, E. K., Coenye, T., Chou, J. H., Barrios, E., de Faria, S. M., et al. (2006). *Burkholderia mimosarum* sp. nov., isolated from root nodules of *Mimosa* spp. from Taiwan and South America. *Int. J. Syst. Evol. Microbiol.* 56, 1847–1851. doi:10.1099/ijs.0.64325-0.

Elliott, G. N., Chen, W. M., Chou, J. H., Wang, H. C., Sheu, S. Y., Perin, L., et al. (2007). *Burkholderia phymatum* is a highly effective nitrogen-fixing symbiont of *Mimosa* spp. and fixes nitrogen *ex planta*. *New Phytol.* 173, 168–180. doi:10.1111/j.1469-8137.2006.01894.x.

Mishra, R. P. N., Tisseyre, P., Melkonian, R., Chaintreuil, C., Miché, L., Klonowska, A., et al. (2012). Genetic diversity of *Mimosa pudica* rhizobial symbionts in soils of French Guiana: investigating the origin and diversity of *Burkholderia phymatum* and other beta-rhizobia. *FEMS Microbiol. Ecol.* 79, 487–503. doi:10.1111/j.1574-6941.2011.01235.x.

Sheu, S. Y., Chou, J. H., Bontemps, C., Elliott, G. N., Gross, E., dos Reis Junior, F. B., et al. (2013). *Burkholderia diazotrophica* sp. nov., isolated from root nodules of *Mimosa* spp. *Int. J. Syst. Evol. Microbiol.* 63, 435–441. doi:10.1099/ijs.0.039859-0.

Sheu, S. Y., Chou, J. H., Bontemps, C., Elliott, G. N., Gross, E., James, E. K., et al. (2012). *Burkholderia symbiotica* sp. nov., isolated from root nodules of *Mimosa* spp. native to north-east Brazil. *Int. J. Syst. Evol. Microbiol.* 62, 2272–2278. doi:10.1099/ijs.0.037408-0.

Vandamme, P., Goris, J., Chen, W.-M., de Vos, P., and Willems, A. (2002). *Burkholderia tuberum* sp. nov. and *Burkholderia phymatum* sp. nov., nodulate the roots of tropical legumes. *Syst. Appl. Microbiol.* 25, 507–512. doi:10.1078/07232020260517634.
