# Supplementary figures and images for: Competition Experiments for Legume Infection Identify Burkholderia phymatum as a Highly Competitive β-Rhizobium
Source: Front Microbiol. 2017 Aug 15;8:1527. doi: 10.3389/fmicb.2017.01527 (PMC5559654; doi:10.3389/fmicb.2017.01527)

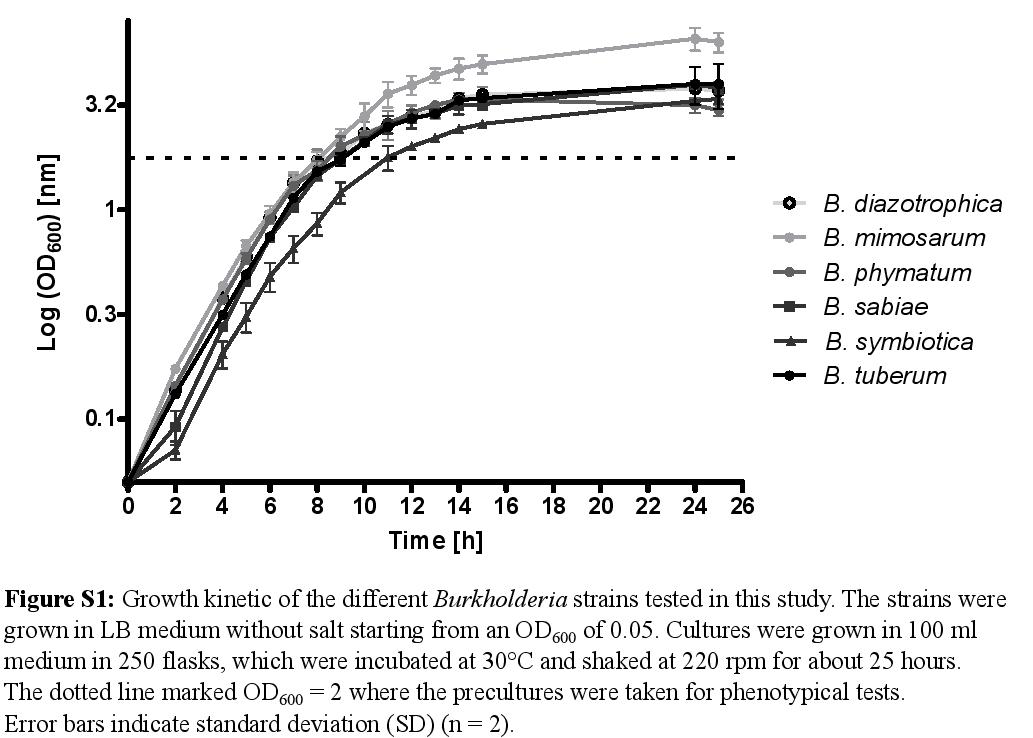

Supplement: Supplementary file 2 [file Image_1.JPEG]

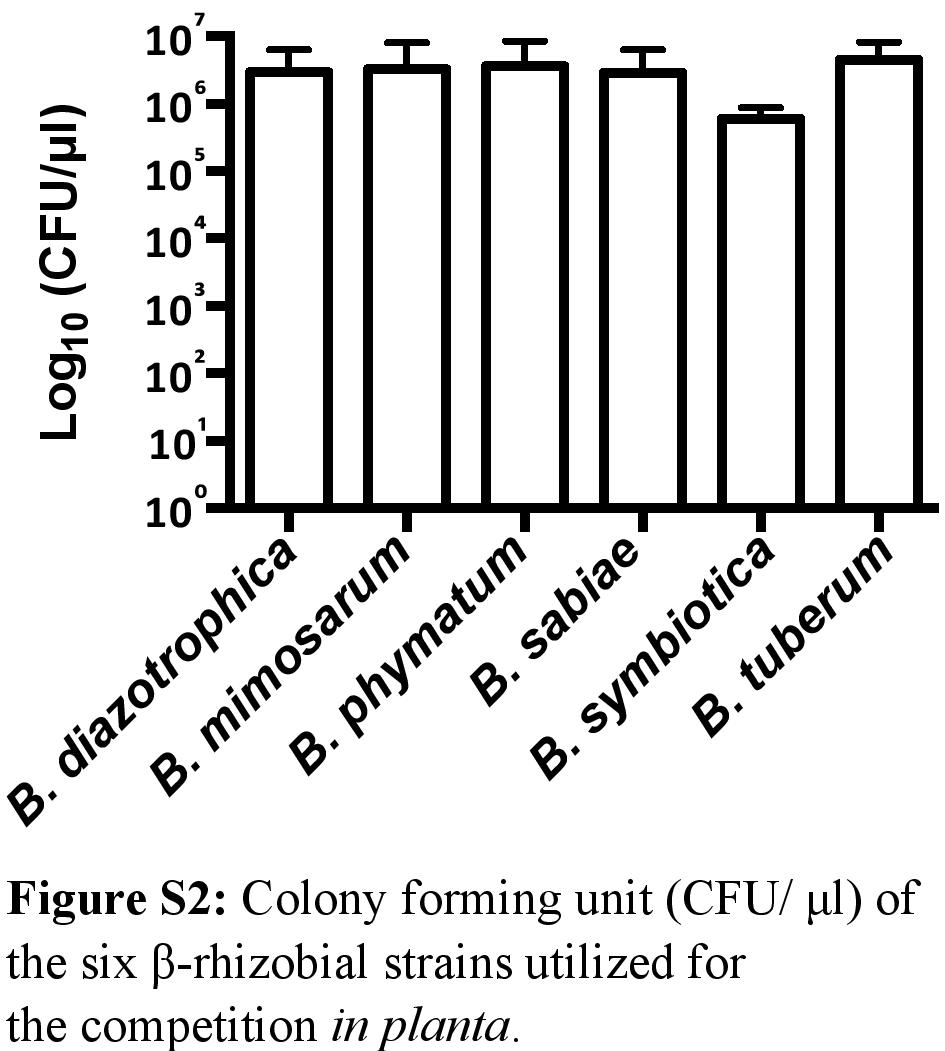

Supplement: Supplementary file 3 [file Image_2.JPEG]

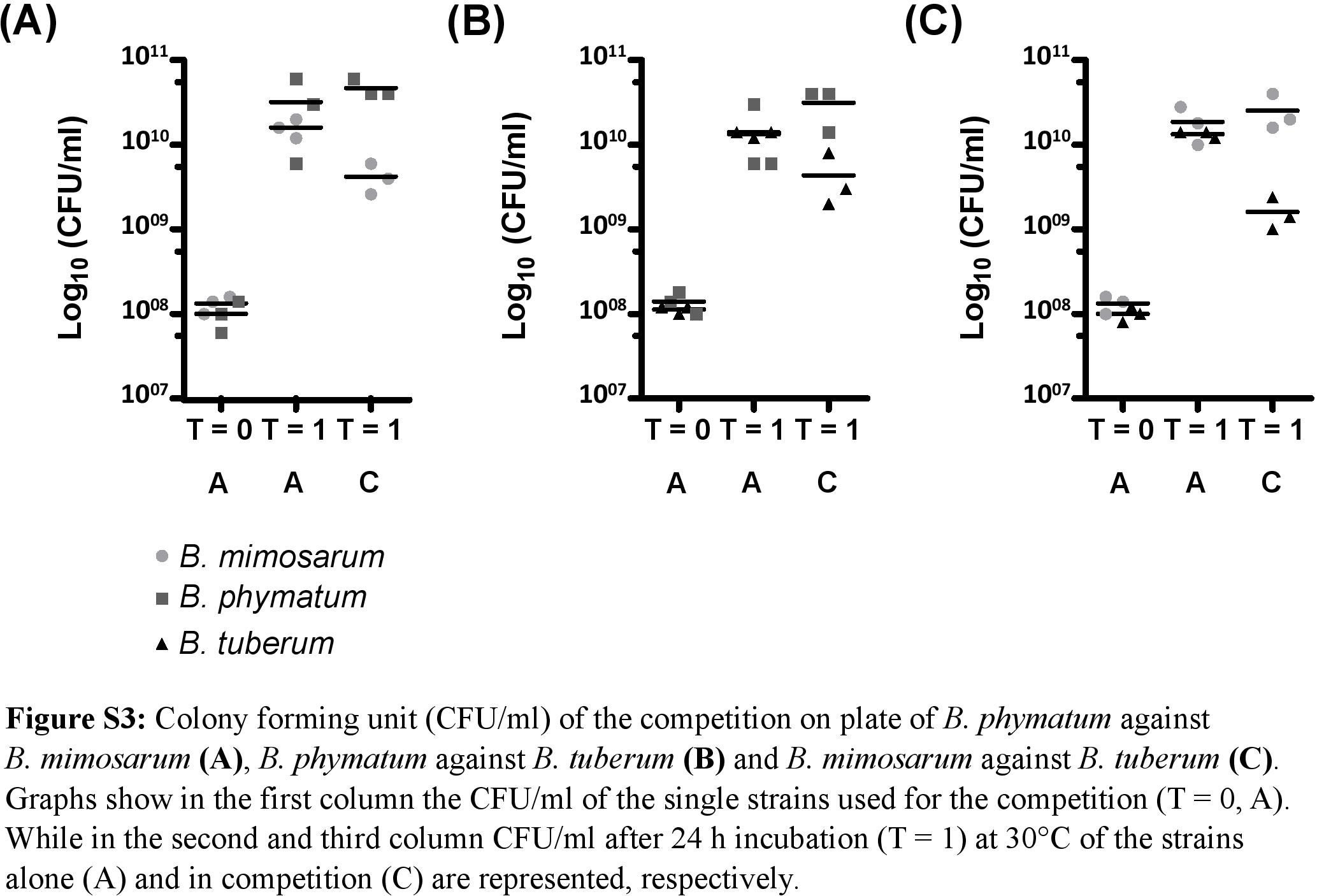

Supplement: Supplementary file 4 [file Image_3.JPEG]
